# High Burden of High-Risk Human Papillomavirus Infections Among Women Living With HIV in Meru, Kenya

**DOI:** 10.7759/cureus.109328

**Published:** 2026-05-21

**Authors:** Celestine K Nyamari, Rachael Gachogo, Anthony K Nyamache, Frank G Onyambu

**Affiliations:** 1 Department of Biochemistry, Microbiology and Biotechnology, Kenyatta University, Nairobi, KEN; 2 Division of Molecular Biology, Centre for Molecular Biosciences and Genomics, Nairobi, KEN; 3 Division of Immunology, Human Pathology, University of Cape Town, Cape Town, ZAF; 4 Department of Medical Laboratory Sciences, Meru University of Science and Technology, Meru, KEN

**Keywords:** cervical cancer, high-risk hpv, hpv genotype distribution, hpv prevalence in kenya, women living with hiv

## Abstract

Background

Cervical cancer remains a major cause of death among women in Kenya and is primarily driven by persistent infection with high-risk human papillomavirus (HR-HPV). HIV infection increases the burden of the disease, as it is associated with higher rates of HPV acquisition and persistence and an increased risk of cervical cancer. Meru County in Kenya is among the regions with a high burden of this cancer, and preventive measures against HR-HPV infections remain limited in this region. It is important to understand the genotype distribution of these infections to guide effective prevention and screening. The prevalence and distribution of circulating HPV genotypes have not been thoroughly characterized, which hinders the development of targeted preventive measures. We therefore aimed to determine the prevalence and genotype distribution of HR-HPV and to assess sociodemographic, behavioral, and clinical factors associated with HR-HPV infection among women living with HIV (WLWHIV) in Meru, Kenya.

Methods

We conducted a cross-sectional study at Meru Teaching and Referral Hospital, Kenya. A total of 303 consenting WLWHIV attending the HIV comprehensive care center at the hospital, aged 18-50 years, were recruited into the study. A structured questionnaire was used to collect data on sociodemographic, health, and behavioral characteristics. High vaginal swabs were collected from the participants and tested for HR-HPV using real-time PCR. All participants were initially screened for HR-HPV, and samples that tested positive for undifferentiated high-risk types were further confirmed by real-time PCR genotyping. Statistical analyses were performed in R (version 4.3.5).

Results

The overall prevalence of HR-HPV was 60.4% (183/303). The most frequent genotypes were HPV 18, detected in 111/303 (36.6%), HPV 45 (96/303, 31.7%), and HPV 16 (37/303, 12.2%). Women who had other high-risk types accounted for 44/303 (14.9%), with HPV 59 detected as the most common genotype (22/44, 50%), followed by HPV 35 (19/44, 43%) and HPV 68 (13/44, 29.5%). Younger women (18-44 years) had more HR-HPV infections compared to older women (45-50 years). Univariate analysis showed an association between HR-HPV and age (p = 0.022), as well as previous cervical cancer screening (p = 0.04), but these associations did not remain significant in multivariate analysis (age: OR = 1.01; 95% CI: 0.91-1.11; p = 0.90; previous cervical cancer screening: OR = 1.21; 95% CI: 0.63-2.32; p = 0.60).

Conclusion

The high prevalence of HR-HPV, dominated by HPV 18, 45, and 16, highlights the importance of strengthening HPV vaccination, screening, and awareness programs among women living with HIV. The higher prevalence observed among younger women further supports the need for targeted early prevention and screening strategies in this population.

## Introduction

Globally, cervical cancer continues to contribute substantially to the cancer burden among women, with an estimated 660,000 new cases and 350,000 deaths reported in 2022 [1]. Among women aged 15-44 years in Kenya, cervical cancer is a leading cause of cancer burden, accounting for approximately 5,236 new cases and 3,211 deaths each year [1]. In Meru County, the incidence of cervical cancer has been increasing, making it the second most common cancer in the region [2]. Mathematical modeling studies indicate that achieving elimination targets in several low- and middle-income countries with high incidence rates and negative trends requires significant enhancement of preventive and curative measures. This includes increasing HPV vaccination and screening programs [3].

HPV is a sexually transmitted infection with over 200 genotypes categorized as low risk, intermediate risk, and HR-HPV [4]. Cervical cancer is primarily caused by persistent HR-HPV infections, with certain genotypes identified as oncogenic, each having varying levels of carcinogenic potential. HPV genotypes with oncogenic potential include 16, 18, 45, 33, 31, 58, 52, 59, 26, 69, 35, 39, 73, 68, 56, 82, 51, 34, 30, 53, 67, 66, 70, and 85 [5]. The majority of cervical cancer cases worldwide are attributed to HPV types 16 and 18 (approximately 70-75%), whereas HPV types 31, 33, 45, 52, and 58 account for a further 15-20%. The remaining causal genotypes account for around 5% of cases and vary by region, for instance, HPV 35, which has a prevalence of 4% in Africa. The eight genotypes associated with the highest risk (HPV 16, 18, 31, 33, 35, 45, 52, and 58) are recognized as the most carcinogenic and are prioritized targets for HPV-based screening strategies and vaccine development. Currently available HPV vaccines differ in the genotypes they target: Cervarix covers types 16 and 18; Gardasil includes types 6, 11, 16, and 18; whereas Gardasil 9 extends coverage to additional types 31, 33, 45, 52, and 58 [5].

HPV is transmitted easily, and the lifetime risk is estimated at 50%-70% for sexually active women [6]. Approximately 90% of infections are cleared naturally within two to three years through cell-mediated immunity, whereas 20% of these infections persist and result in precancerous lesions [7]. Several factors are known to influence the progression of HPV infections to invasive cancer, such as early initiation of sexual activity, coinfections with other sexually transmitted infections, multiple pregnancies, prolonged use of oral contraceptives, immunosuppression due to HIV/AIDS, multiple sexual partners, and tobacco use [6,8,9]. Women living with HIV (WLWHIV) have an increased risk of persistent HPV infections, and progression to cervical cancer occurs approximately 10 years earlier than in those who are HIV negative. Therefore, enhanced HPV screening programs are necessary to help prevent cervical cancer in this population.

The distribution of genotypes, especially those associated with cervical cancer, varies depending on the geographical region, highlighting the need for continuous genotype monitoring [10]. This will guide the development of vaccines and screening strategies tailored to specific regions, particularly in Africa, where HPV distribution differs from global trends [11]. However, genotype-specific distribution among WLWHIV in Meru remains insufficiently characterized, limiting localized prevention strategies.

This study was therefore conducted to determine the prevalence and genotype distribution of HR-HPV among WLWHIV attending Meru Teaching and Referral Hospital in Meru, Kenya, and to assess sociodemographic, behavioral, and clinical factors associated with HR-HPV infection.

## Materials and methods

Study design and setting

A cross-sectional study was conducted between October 2023 and July 2024 at Meru Teaching and Referral Hospital in Meru, Kenya, a level 5 county referral hospital run by the Ministry of Health. The hospital is a major referral center for eastern and northern Kenya and provides both inpatient and outpatient care, including specialized HIV care. This setting ensured access to a broad and diverse patient population for recruitment. All laboratory analyses were performed at the Centre for Molecular Biosciences and Genomics in Nairobi, Kenya.

Sample size and sampling

The Cochran and finite population formulas were used to estimate the sample size, where n is the required sample size, Z is the critical value for the selected confidence interval, p denotes the estimated prevalence, q = 1 − p, and e is the degree of precision. Assuming a 95% confidence interval (Z = 1.96), a prevalence of 27% from a previous study [12], and a precision of 5%, the minimum required sample size was estimated at 303 participants.

Study population

An estimated 303 WLWHIV aged 18-50 years receiving care at the Meru Teaching and Referral Hospital HIV Comprehensive Care Centre were recruited using systematic sampling, where every third eligible woman who visited the clinic was invited to participate. The sampling interval was determined based on the estimated clinic attendance during the study period and the required sample size. If a selected participant declined participation or did not meet the eligibility criteria, the subsequent eligible woman was approached for recruitment. This approach minimized selection bias and provided eligible participants with an equal opportunity to be included in the study. Women who had been previously diagnosed with cervical cancer, women with active vaginal bleeding, pregnant women, and HIV-negative women visiting the facility for HIV testing were excluded from the study.

Sample collection

The Evalyn Brush® (Rovers Medical, Netherlands) was used by study participants to self-collect high vaginal samples following the manufacturer’s instructions. After sample collection, the brush was returned to the original packaging and transported at room temperature to the Centre for Molecular Biosciences and Genomics Laboratory for HPV testing and genotyping. Samples were stored at -20 °C until analysis.

DNA extraction

Viral DNA was isolated from the Evalyn Brush using the GeneProof Pathogen Free DNA Isolation Kit (GeneProof, Czech Republic). All procedures were performed in accordance with the manufacturer’s instructions. Briefly, the brush was suspended in 1 mL of nuclease-free water, from which 200 µL was used as the starting material for DNA extraction. The cells were lysed in chaotropic salts and proteinase K, and incubation at 70 °C for 30 minutes enabled lysis, allowing the released DNA to bind to the silica matrix in the spin columns. This was followed by ethanol precipitation and elution in nuclease-free water, resulting in pure DNA suitable for PCR.

High-risk HPV screening and genotyping

The GeneProof Human Papillomavirus Screening Kit (GeneProof, Czech Republic) was used as the primary screening assay for HR-HPV detection. The kit detects 24 high-risk types, including 16, 18, 26, 30, 31, 33, 34, 35, 39, 45, 51, 52, 53, 56, 58, 59, 66, 67, 68, 69, 70, 73, 82, and 97, with specific differentiation for HPV 16, 18, and 45, while the remaining high-risk types are collectively detected as other HR-HPV. The kit targets conserved regions within the E1/E2 region of the HPV genome, with the exact primer and probe sequences proprietary to the manufacturer. Each reaction consisted of 5 µL of extracted DNA combined with 15 µL of master mix, yielding a final volume of 20 µL. The amplification protocol included an initial uracil-N-glycosylase (UNG) decontamination step at 34 °C for two minutes, followed by DNA denaturation at 95 °C for 10 minutes. This was followed by 45 amplification cycles comprising denaturation at 95 °C for five seconds, annealing at 60 °C for 40 seconds, and extension at 72 °C for 20 seconds. Fluorescence signals were detected across multiple channels: FAM for HR-HPV, Cy5 for HPV 16, Texas Red for HPV 18, Quasar 705 for HPV 45, and HEX as the internal control. All reactions were performed using a Bio-Rad CFX96 Real-Time PCR system.

Samples positive for HR-HPV types other than HPV 16, 18, and 45 on the GeneProof assay were subsequently analyzed using the AmpFire High-Risk HPV Genotyping Kit (Attila BioSystems, Sunnyvale, CA) to allow further genotype-specific characterization of additional HR-HPV types. The kit is based on isothermal amplification of HR-HPV target regions within the HPV genome, such as E6 and E7, using specific primers and probes, with real-time fluorescence detection of HPV 16, 18, 31, 33, 35, 39, 45, 51, 52, 53, 56, 58, 59, 66, and 68. To lyse the cells, 19.5 µL of purified DNA was added to a tube containing 0.5 µL of 40× lysis buffer. The mixture was vortexed, briefly spun down, and incubated at 95 °C for 20 minutes. Multiplex reactions were prepared using four primer mixes supplied with the kit. The master mix for each of the four reactions consisted of 10 µL of reaction mix (containing buffer, enzyme mix, dNTPs, and Mg²⁺) and 10 µL of the primer mix, which included genotype-specific primers and probes proprietary to the manufacturer. The PCR involved 60 cycles of denaturation and extension, both at 60 °C for 30 seconds with fluorescence acquisition. Primer mix 1 detected HPV31 (FAM), HPV51 (HEX), HPV39 (ROX), and HPV16 (Cy5). Primer mix 2 detected HPV35 (FAM), HPV68 (HEX), HPV18 (ROX), and HPV59 (Cy5). Primer mix 3 detected HPV33 (FAM), internal control (HEX), HPV66 (ROX), and HPV45 (Cy5). Primer mix 4 detected HPV58 (FAM), HPV56 (HEX), HPV53 (ROX), and HPV52 (Cy5).

Statistical analysis

Statistical analyses were performed using R (version 4.3.5, R Foundation for Statistical Computing, Vienna, Austria). Descriptive, bivariate, and multivariable analyses were conducted to examine associations between HR-HPV and sociodemographic, behavioral, and health factors. Categorical variables are reported as frequencies and percentages, whereas continuous variables are summarized using medians and interquartile ranges (IQR). Associations between categorical variables were evaluated using Pearson’s chi-square test or Fisher’s exact test where appropriate, whereas the Wilcoxon rank-sum test was used for continuous variables. Statistical significance was defined as a p-value of <0.05. Multivariable logistic regression analysis was conducted to identify independent predictors of HPV positivity, with adjustment for relevant covariates. Findings are presented as adjusted odds ratios (aORs) with 95% confidence intervals (CIs). Variables included in the multivariable logistic regression model were selected based on evidence from previous literature on factors associated with HR-HPV infection. Missing data were minimal, and complete-case analysis was performed, whereby participants with missing data for variables included in a given analysis were excluded from that analysis.

Ethical approval

Ethical clearance was obtained from the Meru University Institutional Ethics Review Committee (MIRERC003/2023). The research license was obtained from the National Commission for Science, Technology and Innovation (NACOSTI/P/24/34603). Written informed consent was obtained from all participants prior to enrollment. Confidentiality was maintained through the use of de-identified data and secure data-handling procedures.

## Results

Baseline characteristics of study participants stratified by HR-HPV status

The majority of the 303 participants recruited were aged 30-44 years (54%), with a median age of 40 years (IQR 34-46). A total of 183 (60.4%) had completed primary school education, with most being self-employed (70.3%, n = 213) and earning a monthly income of less than Ksh 20,000. A total of 91 (30%) participants reported sexual debut between the ages of 16 and 19 years, 57.1% (n = 173) reported contraceptive use, and 189 (62.4%) had one to two children. Almost half of the participants were married or cohabiting (46.9%), and most reported no history of smoking (99.7%), alcohol consumption (99.0%), or illicit substance use (99.3%). Younger age and previous cervical cancer screening were associated with HR-HPV positivity in univariate analysis (age: p = 0.022; previous cervical cancer screening: p = 0.04). Participant characteristics stratified by HR-HPV status are presented in Table 1.

**Table 1:** Characteristics of study participants stratified by HR-HPV status ^1^Continuous variables are presented as median (IQR) and categorical variables as n (%). ^2^P-values were calculated using the Wilcoxon rank-sum test for continuous variables and Pearson’s chi-squared test or Fisher’s exact test for categorical variables. STI, sexually transmitted infection; ART, antiretroviral therapy; VIA/VILI, visual inspection with acetic acid/Lugol’s iodine; HR-HPV, high-risk human papillomavirus.

Characteristic	HPV-Negative^1^ (N = 120)	HPV-Positive^1^ (N = 183)	Overall^1^ (N = 303)	P-value^2^
Age, years	42 (34, 47)	38 (33, 45)	40 (34, 46)	0.022
Age group				
18-29	16 (13.3%)	30 (16.4%)	46 (15.2%)	
30-44	57 (47.5%)	106 (57.9%)	163 (53.8%)	
45-50	47 (39.2%)	47 (25.7%)	94 (31.0%)	
Education level				0.7
Primary	77 (64.2%)	106 (57.9%)	183 (60.4%)	
Secondary school	35 (29.2%)	62 (33.9%)	97 (32.0%)	
College	6 (5.0%)	12 (6.6%)	18 (5.9%)	
None	2 (1.7%)	3 (1.6%)	5 (1.7%)	
Monthly income				0.3
0-20,000	112 (93.3%)	164 (89.6%)	276 (91.1%)	
21,000-35,000	8 (6.7%)	19 (10.4%)	27 (8.9%)	
Employment status				0.5
Employed	17 (14.2%)	31 (16.9%)	48 (15.8%)	
Not employed	14 (11.7%)	28 (15.3%)	42 (13.9%)	
Self-employed	89 (74.2%)	124 (67.8%)	213 (70.3%)	
Marital status				0.3
Divorced	2 (1.7%)	10 (5.5%)	12 (4.0%)	
Married/cohabiting	58 (48.3%)	84 (45.9%)	142 (46.9%)	
Single	49 (40.8%)	78 (42.6%)	127 (41.9%)	
Widowed	11 (9.2%)	11 (6.0%)	22 (7.3%)	
Parity				>0.9
1-2 times	76 (63.3%)	113 (61.7%)	189 (62.4%)	
3-4 times	42 (35.0%)	67 (36.6%)	109 (36.0%)	
5+ times	1 (0.8%)	2 (1.1%)	3 (1.0%)	
None	0 (0%)	1 (0.5%)	1 (0.3%)	
Contraceptive use				0.3
No	46 (38.3%)	83 (45.4%)	129 (42.6%)	
Yes	73 (60.8%)	100 (54.6%)	173 (57.1%)	
Length of contraceptive use				0.2
0-2 years	2 (1.7%)	1 (0.5%)	3 (1.0%)	
3-4 years	11 (9.2%)	26 (14.2%)	37 (12.2%)	
5+ years	60 (50.0%)	73 (39.9%)	133 (43.9%)	
Age at first contraceptive use				0.9
16-18 years	10 (8.3%)	15 (8.2%)	25 (8.3%)	
More than 18 years	65 (54.2%)	91 (49.7%)	156 (51.5%)	
Age of sexual debut				0.078
16-19 years	29 (24.2%)	62 (33.9%)	91 (30.0%)	
Above 20 years	90 (75.0%)	121 (66.1%)	211 (69.6%)	
Years with current sexual partner				0.7
Long-term	25 (20.8%)	31 (16.9%)	56 (18.5%)	
Medium-term	22 (18.3%)	38 (20.8%)	60 (19.8%)	
Short-term	11 (9.2%)	16 (8.7%)	27 (8.9%)	
Condom use				0.2
Always	24 (20.0%)	52 (28.4%)	76 (25.1%)	
Never	30 (25.0%)	35 (19.1%)	65 (21.5%)	
Sometimes	65 (54.2%)	96 (52.5%)	161 (53.1%)	
Previous cervical cancer screening				0.04
Pap smear	68 (56.7%)	82 (44.8%)	150 (49.5%)	
VIA/VILI	51 (42.5%)	100 (54.6%)	151 (49.8%)	
Abnormal results after screening				0.7
No	118 (98.3%)	179 (97.8%)	297 (98.0%)	
Yes	1 (0.8%)	4 (2.2%)	5 (1.7%)	
Time to ART initiation				0.3
>36 months	23 (19.2%)	28 (15.3%)	51 (16.8%)	
0 months	66 (55.0%)	118 (64.5%)	184 (60.7%)	
1-12 months	12 (10.0%)	18 (9.8%)	30 (9.9%)	
13-36 months	19 (15.8%)	18 (9.8%)	37 (12.2%)	

High-risk HPV prevalence and genotype distribution

**Figure 1:**
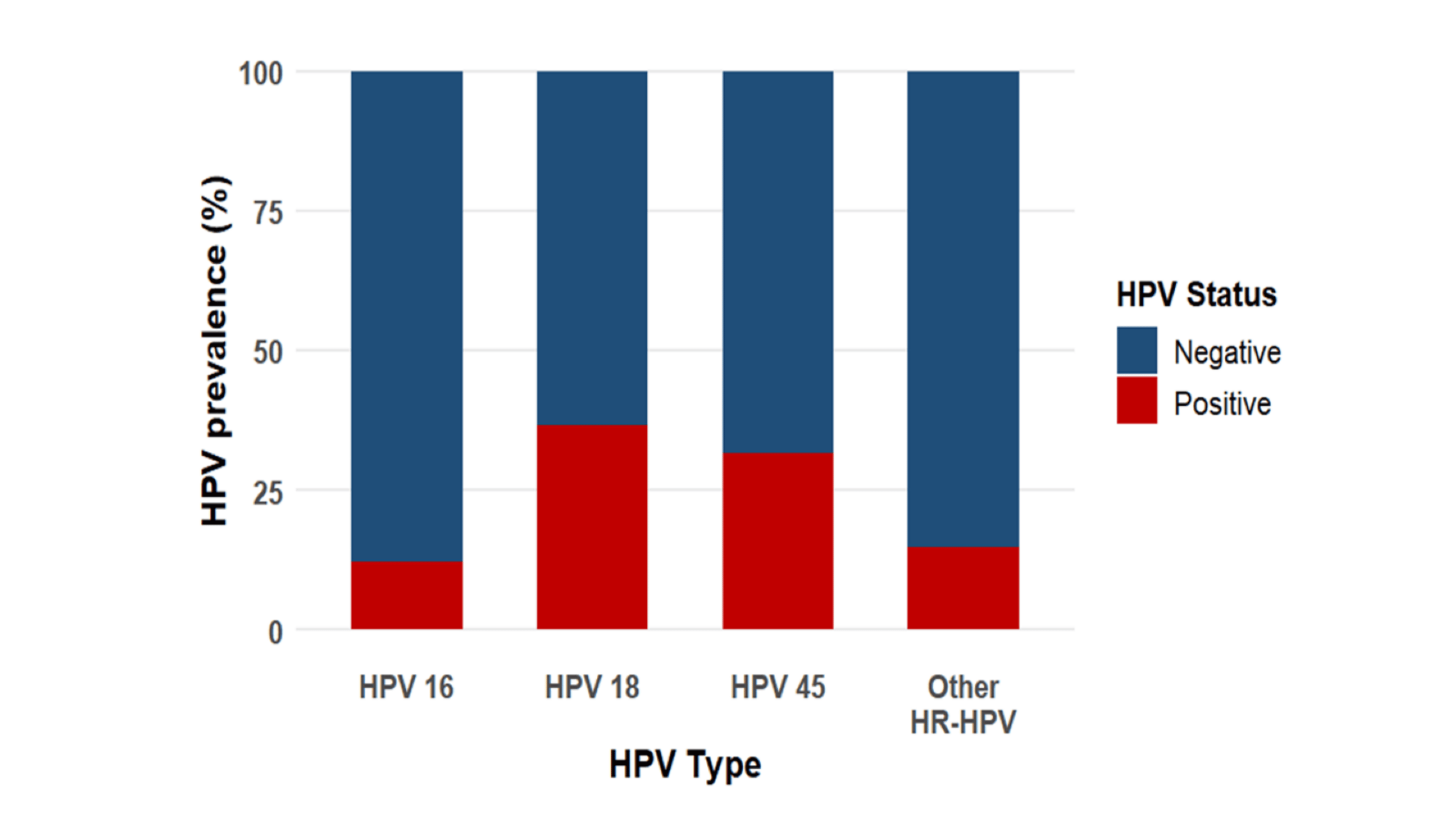
Distribution of HR-HPV genotypes among study participants HPV, human papillomavirus; HR-HPV, high-risk human papillomavirus.

The overall HR-HPV prevalence was 60.4% (183/303). A total of 111/303 (36.6%) participants had HPV 18, 96/303 (31.68%) had HPV 45, and 37/303 (12.2%) had HPV 16. Other HR-HPV types accounted for 44/303 (14.85%) of the infections (Figure 1). 

Among the samples classified as other HR-HPV types (n = 44), a total of 15 genotypes were identified. The most common genotypes were HPV 59, detected in 22/44 (50%); HPV 35, in 19/44 (43%); and HPV 68, in 13/44 (29.5%). Other genotypes were also detected, including HPV 31 and HPV 51 (each in 8/44, 18.2%); HPV 52 (7/44, 15.9%); HPV 39, HPV 56, HPV 58, and HPV 66 (each in 3/44, 6.8%); HPV 33 (2/44, 4.5%); and HPV 53 (1/44, 2.3%).

HPV prevalence across age groups

**Figure 2:**
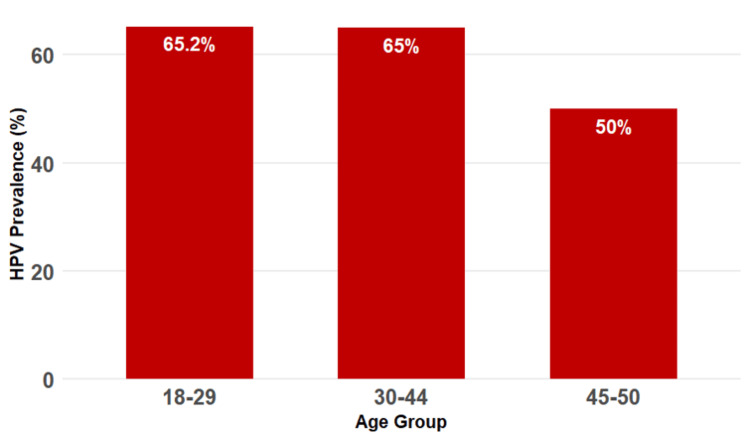
Age-specific prevalence of HR-HPV among study participants HPV, human papillomavirus; HR-HPV, high-risk human papillomavirus.

Prevalence was similar among women aged 18-29 years (65.2%, n = 30/46) and 30-44 years (65.0%, n = 106/163) but was slightly lower among women aged 45-50 years (50.0%, n = 47/94). Overall, the highest prevalence was observed among women aged 30-44 years, who represented the largest proportion of the study population (Figure 2). 

HPV co-infection patterns

**Figure 3:**
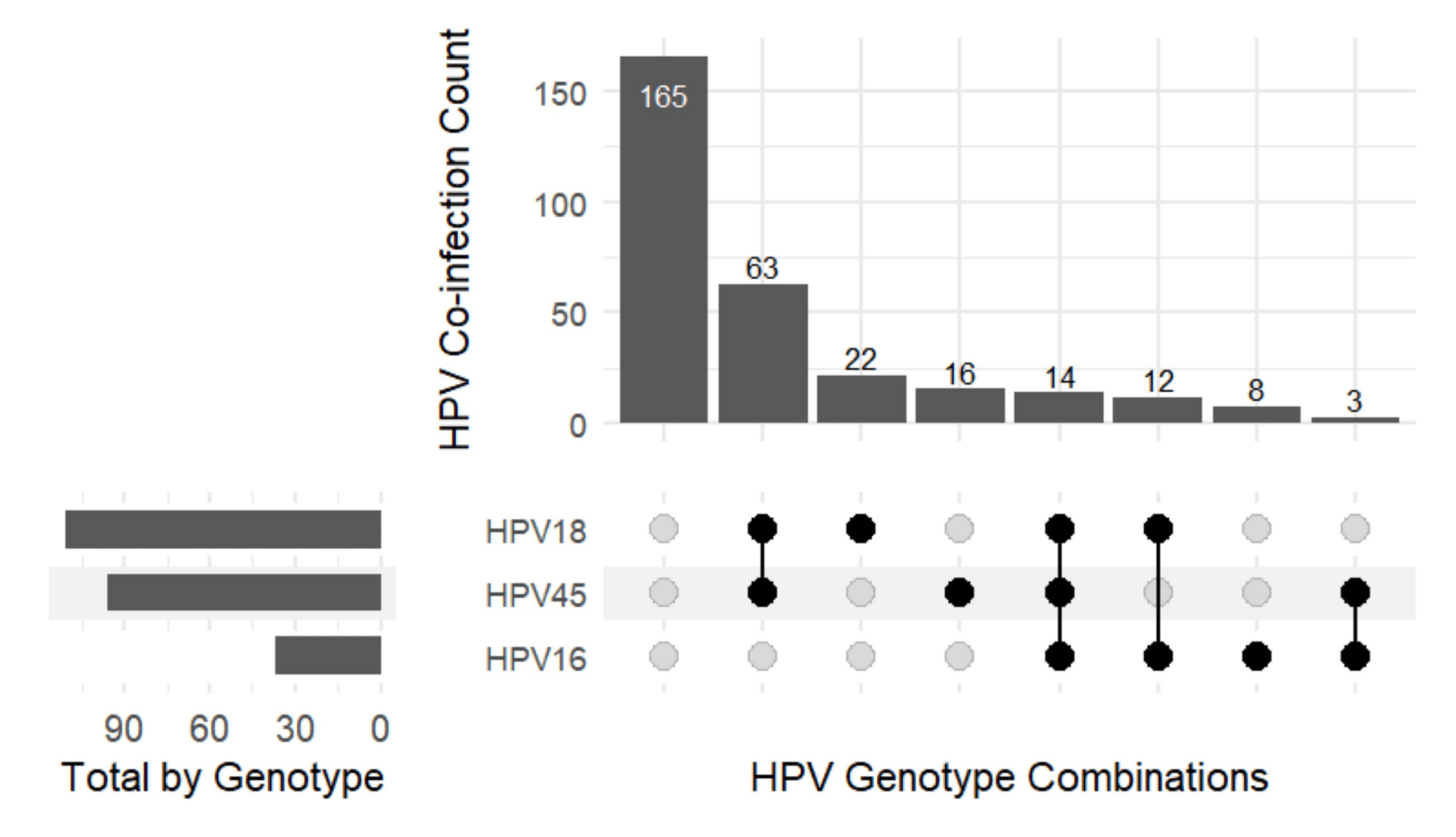
Upset plot of co-infection patterns among HPV 16, 18, and 45 HPV, human papillomavirus.

The most common coinfection in our sample was HPV 18 and HPV 45 coinfection, observed in 63 women (20.8%). A total of 14 women (4.6%) were coinfected with HPV 16, 18, and 45, whereas 12 (4.0%) were coinfected with HPV 16 and HPV 18, and three (1.0%) with HPV 16 and HPV 45 (Figure 3). 

Coinfection was also common among participants with other HR-HPV infections. The most frequent coinfections were between HPV 45 and HPV 59 and between HPV 35 and HPV 59 (16 samples each), followed by HPV 16 and HPV 45 (14 samples). HPV 59 exhibited the highest overall coinfection frequency, often co-occurring with HPV 45 and HPV 35 (16 samples each) and with HPV 16 and HPV 68 (11 samples each).

Association between participant characteristics and HR-HPV status

A significant association was observed between age and HPV positivity in univariate analysis. The median age for HPV-positive participants was 38 years (IQR, 33-45), whereas the median age for HPV-negative participants was 42 years (IQR, 34-47), with a significant association between age and HPV status (p = 0.022) (Table 1). We also noted a statistically significant association between previous cervical cancer screening and HPV status (p = 0.04), which may reflect bias in healthcare access.

Multivariate analysis of factors associated with HPV infections

Multivariable logistic regression analysis did not identify any participant characteristics significantly associated with HPV infection (Table 2). Although age and previous cervical cancer screening were significant in the univariate analysis, these associations were not retained after adjusting for other covariates. There was no significant association between age and HPV infection (aOR = 1.01; 95% CI: 0.91-1.11; p = 0.90). Similarly, previous cervical cancer screening showed no significant association with HPV status (aOR = 1.21; 95% CI: 0.63-2.32; p = 0.60). Women who reported sexual debut after 20 years of age showed lower odds of HPV infection compared to those with sexual debut between 16 and 19 years (aOR = 0.44; 95% CI: 0.18-1.04), although the association did not reach statistical significance (p = 0.068). 

**Table 2:** Multivariate logistic regression analysis of variables associated with HPV infections HPV, human papillomavirus; aOR, adjusted odds ratio; CI, confidence interval; VIA/VILI, visual inspection with acetic acid/visual inspection with Lugol’s iodine; STI, sexually transmitted infections.

Characteristic	aOR	95% CI	p-value
Age	1.01	0.91, 1.11	0.9
Number of children (parity)			
1-2 times	—	—	
3 or more times	1.71	0.85, 3.55	0.14
Contraceptive use			
No	—	—	
Yes	0.42	0.06, 2.13	0.3
Marital status			
Divorced	—	—	
Married/cohabiting	0.43	0.06, 2.12	0.3
Single	0.43	0.06, 2.09	0.3
Widowed	1.21	0.12, 10.8	0.9
Age at sexual debut			
16-19 years	—	—	
Above 20 years	0.44	0.18, 1.04	0.068
Age started contraceptives			
16-18 years	—	—	
More than 18 years	1.19	0.42, 3.40	0.7
Condom use (past 6 months)			
Always	—	—	
Never	0.93	0.32, 2.70	0.9
Sometimes	0.96	0.37, 2.45	>0.9
Previous cervical cancer screening			
Pap smear	—	—	
VIA/VILI	1.21	0.63, 2.32	0.6
History of STIs			
No	—	—	
Yes	1.49	0.23, 12.0	0.7

## Discussion

This study investigated the prevalence and genotypes of HR-HPV and risk factors linked to these infections among a cohort of women living with HIV in Meru, Kenya. Over half of the participants tested positive for HR-HPV, with more infections observed among younger women. HPV 18 and HPV 45 were the most common genotypes detected in this cohort.

The prevalence of HR-HPV globally varies widely, with the burden substantially higher among WLWHIV compared to the general female population. Studies in Sub-Saharan Africa report prevalence ranging from 30%-60% among WLWHIV and 20%-30% among the general population [13-15]. In Kenya, studies report similar trends. A systematic review reported a pooled HR-HPV prevalence of 64% among WLWHIV [16]. Another study conducted across Eastern Kenya also reported a high prevalence among this demographic [17]. Our study reported a prevalence of 60.4%, consistent with the findings from these previous studies. This could be attributed to HIV affecting immune competence, reducing the ability to clear infections and promoting viral persistence at the cervical mucosa [18]. Overall, our findings confirm the high burden of HR-HPV infections among WLWHIV.

HPV 18, HPV 45, and HPV 16 were the most common genotypes observed in our cohort. Comprehensive genotyping on a subset of samples initially classified as other high-risk types revealed HPV 59, HPV 35, and HPV 68 as the most frequent, indicating the need for genotyping to detect additional genotypes not identified by screening assays. Worldwide, the distribution of HPV genotypes is dominated by types 16 and 18, which are responsible for nearly 70% of cervical cancer cases [19]. In Sub-Saharan Africa, variations in detected genotypes have been observed, with HPV 16, 52, 18, and 35 reported as the most common [15]. In Kenya, HPV 16, 18, 35, 52, and 45 are frequently observed [14,20]. Other studies have reported a high prevalence of HPV 35, observed as commonly as HPV 18 [21,22]. The variations observed in genotype distribution may reflect geographical differences, participant characteristics, immune status, and genotyping tests that detect a wider range of HPV types. The similarities in our findings suggest a consistent pattern of specific genotypes among WLWHIV. Our findings are also in line with previous literature showing that WLWHIV have a broad spectrum of HR-HPV genotypes. Some of the genotypes identified in our study, such as HPV 35 and HPV 68, are not covered by currently available vaccines. This highlights the need for continuous surveillance and supplementation of current vaccines to include these genotypes.

We observed HR-HPV coinfections in this cohort, with HPV 18 and HPV 45 coinfection being the most common. This could be due to the close phylogenetic relationship between these genotypes, as they both belong to the Alpha-7 species. The observed coinfections could also be a result of shared transmission dynamics. Other studies in Sub-Saharan Africa have shown frequent coinfections among WLWHIV compared to HIV-negative women, with HPV 18 and HPV 45 commonly detected either alone or in combination with other high-risk genotypes, including HPV 16 [14,15]. HR-HPV coinfections are concerning because they contribute to the persistence of oncogenic HPV types, thereby elevating the likelihood of high-grade lesions and rapid progression to cancer [23].

Univariate analyses showed a higher prevalence of HR-HPV positivity among younger women, although the association was not significant after accounting for other characteristics, indicating that age alone is not an independent predictor of HR-HPV infections. This finding is consistent with previous studies reporting high prevalence among younger women and may reflect higher exposure to new HPV infections in younger age groups before immune-mediated clearance of transient infections. Other studies report HR-HPV prevalence ranging from 40%-70% in women younger than 40 years [24-26]. Another study showed a bimodal HR-HPV prevalence distribution, with a peak prevalence observed among younger women (40%) and a second peak in postmenopausal women (30%) [27]. These findings highlight the importance of age-targeted cervical cancer screening and prevention strategies that also consider other relevant risk factors.

Study limitations

The cross-sectional study design used does not allow inference of causality between risk factors and HR-HPV infection. Additionally, the screening assay used for all 303 participants detected 24 HR-HPV types but differentiated only HPV 16, HPV 18, and HPV 45, whereas the remaining types were grouped as other HR-HPVs. These other HR-HPV samples were further tested using a comprehensive genotyping assay that detected a wider range of genotypes. As a result, the full genotype distribution of all HR-HPV types was not determined for the entire study population, which may have led to underestimation of the prevalence of specific genotypes. Additionally, HIV-related clinical variables such as CD4 count, viral load, antiretroviral therapy (ART) regimen, duration of ART use, and degree of immune suppression were not available for inclusion in the analysis, limiting assessment of their potential association with HR-HPV infection. Furthermore, the single-center study design and systematic sampling approach may limit the generalizability of the findings to other populations of women living with HIV. Limited variability in some self-reported behavioral factors, such as smoking and alcohol use, may also have reduced the ability to assess their association with HR-HPV infection.

## Conclusions

This study reports a high burden of HR-HPV infection among women living with HIV in Meru, Kenya, with HPV 18, HPV 45, and HPV 16 being the predominant genotypes identified. Additional genotypes not covered by current vaccines, including HPV 35, 59, and 68, were also detected, although these findings should be interpreted cautiously because comprehensive genotyping was not performed for all HR-HPV-positive samples. The findings highlight the diverse HR-HPV genotype distribution in this population and the importance of continued surveillance to inform cervical cancer prevention and screening strategies.

Although no independent risk factors were identified in multivariable analysis, younger women had a higher prevalence of HR-HPV infection, and coinfections were observed among some participants. The absence of key HIV-related clinical variables may have limited the assessment of immunological influences on HR-HPV infection. Overall, these findings support continued efforts to strengthen HPV vaccination, expand genotype-inclusive screening approaches, and improve surveillance systems to better characterize circulating HR-HPV genotypes among women living with HIV in this setting.
